# Time series analysis and forecasting of chlamydia trachomatis incidence using surveillance data from 2008 to 2019 in Shenzhen, China

**DOI:** 10.1017/S0950268820000680

**Published:** 2020-03-17

**Authors:** R. X. Weng, H. L. Fu, C. L. Zhang, J. B. Ye, F. C. Hong, X. S. Chen, Y. M. Cai

**Affiliations:** 1 Department of STD control and prevention, Shenzhen Center for Chronic Disease Control, Shenzhen, Guangdong Province 518020, China; 2 Department of Epidemiology and Health Statistics, XiangYa School of Public Health, Central South University, Changsha, Hunan Province 410078, China; 3 Chinese Academy of Medical Sciences & Peking Union Medical College Institute of Dermatology, Nanjing, China; 4 National Center for STD Control, China Center for Disease Control and Prevention, Nanjing, China

**Keywords:** Chlamydia trachomatis, forecasting, SARIMA

## Abstract

Chlamydia trachomatis (CT) infection has been a major public health threat globally. Monitoring and prediction of CT epidemic status and trends are important for programme planning, allocating resources and assessing impact; however, such activities are limited in China. In this study, we aimed to apply a seasonal autoregressive integrated moving average (SARIMA) model to predict the incidence of CT infection in Shenzhen city, China. The monthly incidence of CT between January 2008 and June 2019 in Shenzhen was used to fit and validate the SARIMA model. A seasonal fluctuation and a slightly increasing pattern of a long-term trend were revealed in the time series of CT incidence. The monthly CT incidence ranged from 4.80/100 000 to 21.56/100 000. The mean absolute percentage error value of the optimal model was 8.08%. The SARIMA model could be applied to effectively predict the short-term CT incidence in Shenzhen and provide support for the development of interventions for disease control and prevention.

## Introduction

Chlamydia trachomatis (CT) is one of the most prevalent sexually transmitted diseases worldwide. According to the updated estimates from the World Health Organization, there were 127.2 million new CT cases among people aged 15–49 years in 2016 [1]. Infection of CT, if not treated properly and promptly, can result in serious sequelae, such as pelvic inflammatory disease, ectopic pregnancy, tubal infertility and chronic pelvic pain in women and non-gonococcal urethritis, epididymitis and infertility in men [2–5]. Additionally, evidence suggests that CT infections contribute to the transmission of HIV and human papillomavirus-associated cervical carcinoma development [6, 7]. It is estimated that the lifetime direct medical costs for chlamydia alone were ~$516.7 million in the United States, which is a great burden to individuals and society [8]. Therefore, monitoring and prediction of epidemic status and trends of CT infections are critical for precision planning of CT control programme, appropriate allocation of available resources and accurate evaluation of implementation outcomes.

Presently, numerous useful mathematical and statistical methods together with their corresponding technologies (e.g. software tools) have been developed and widely applied in diseases forecasting. Among them, time series analysis is one of the quantitative methods which can effectively predict the future incidence of communicable diseases and epidemiological trends using previously observed data and time variables [9, 10]. This analysis deals with time-dependent variables with an advantage of being not necessary to consider the influence of intricate factors [11, 12]. As a kind of time series analysis [13], the seasonal autoregressive integrated moving average (SARIMA) model has been increasingly favoured and successfully used in the prediction of communicable diseases, such as dengue [14], tuberculosis [15], mumps [12] and others [10, 16, 17]. The SARIMA model has good performance in short-term prediction and is easy to implement [18]. Additionally, the SARIMA model decomposes time series into trend, seasonal and residual components, which can improve prediction accuracy.

Shenzhen is a modern city with a large population density and floating population. The reported incidence of CT infection in Shenzhen was 171.23/100 000 in 2014, which was the highest in the entire province and much higher than the national level [19, 20]. Obviously, CT infection remains a major public health issue in Shenzhen, and the prevention and control of CT infection faces difficulties and challenges. In order to have a better understanding of the magnitude of future CT burden in the general population and have early detection of CT outbreaks, advanced prediction of CT epidemic in Shenzhen is needed. In this study, based on surveillance data from January 2008 to June 2019, we designed a SARIMA model to forecast the temporal trends of CT incidence in Shenzhen, China.

## Methods

### Data collection

CT was included in the surveillance system since 2008, the data on CT was available from then on. Cases with a diagnosis of CT at health facilities in surveillance sites in China should be reported to the Chinese National Disease Surveillance Reporting and Management System for Disease Control and Prevention within 24 h by hospital physicians. The observed monthly cases of CT infection in the study period were extracted from the Chinese National Disease Surveillance Reporting and Management System and the population data was collected from Shenzhen Statistics Bureau. In this study, we obtained the incidence time series of CT from January 2008 to June 2019. According to national guidelines [21], CT is defined as a clinically compatible case characterised by the positivity of cell culture, antigen detection, microscopy or nucleic acid amplification test.

### Construction of the SARIMA model

The model fitting process was divided into two parts: a training period for constructing SARIMA models and a hold-out period for validation. Corresponded to the analytical phase, the data on CT incidence was divided into a training dataset from January 2008 to June 2018 and a hold-out dataset from July 2018 to June 2019. The application of SARIMA based on the Box and Jenkins was approached [18].

The equation of the seasonal autoregressive integrated moving average SARIMA model:1
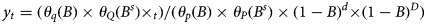


where *θ_q_*(*B*) and *θ_Q_*(*B^s^*) are the moving average (MA) operator and seasonal MA operator, respectively; *θ_p_*(*B*) and *θ_P_*(*B^s^*) are the autoregressive operator (AR) and seasonal AR operator, respectively. *B* and *y_t_* are the backward shift operator and the dependent variable, respectively. *ɑ_t_* represents white noise. *d* and *D* are the order of non-seasonal and seasonal difference, respectively. The expression of the SARIMA model could be like this: SARIMA (*p*, *d*, *q*) × (*P*, *D*, *Q*)*_S_*, where *p*, *d* and *q* represent the order of AR, the degree of difference and the order of MA, respectively; *P*, *D* and *Q* represent the order of seasonal AR, the seasonal integration and the order of seasonal MA, respectively and *s* represents the lengths of seasonal period in months [18].

There are three steps in the SARIMA modelling procedure: identification, estimation and diagnosis [22]. Before constructing SARIMA models, the time series should be checked by the Ljung–Box test for white noise. In addition, the series should be stationary and its stationarity was tested by the Augmented Dickey–Fuller (ADF) method [23]. If the time series is not stationary, we performed the regular difference or the seasonal difference for the SARIMA model to ensure the stationarity of the time series, then *d* and *D* were confirmed by the above difference. The orders of *p*, *q*, *P* and *Q* were identified by using the autocorrelation functions (ACF) and the partial autocorrelation functions (PACF) in the differenced series. The optimum model with good performing was selected according to the lowest value of either Akaike Information Criterion (AIC) or the Schwartz Bayesian Criterion (SBC) [24]. The conditional least square method was used to estimate parameters after identifying the optimal model and the *t* test was used to test parameters [25]. Next, white noise and residuals were checked by the Ljung–Box test [26, 27], and the *Q–Q* plot was used to check the normality of residuals. Finally, we forecasted the monthly CT incidence in the hold-out period using the optimal model. The mean absolute percentage error (MAPE) between predicted values and the actual values was used to assess the accuracy of the SARIMA model.

### Ethical review

The individual data without identifiable personal information were from the web-based Chinese National Disease Surveillance Reporting and Management System for Disease Control and Prevention. The current study was approved by the Ethics Committee of Shenzhen Center for Chronic Disease Control (Approval No. 20180206).

## Results

### Overall trends

**Fig. 1.**
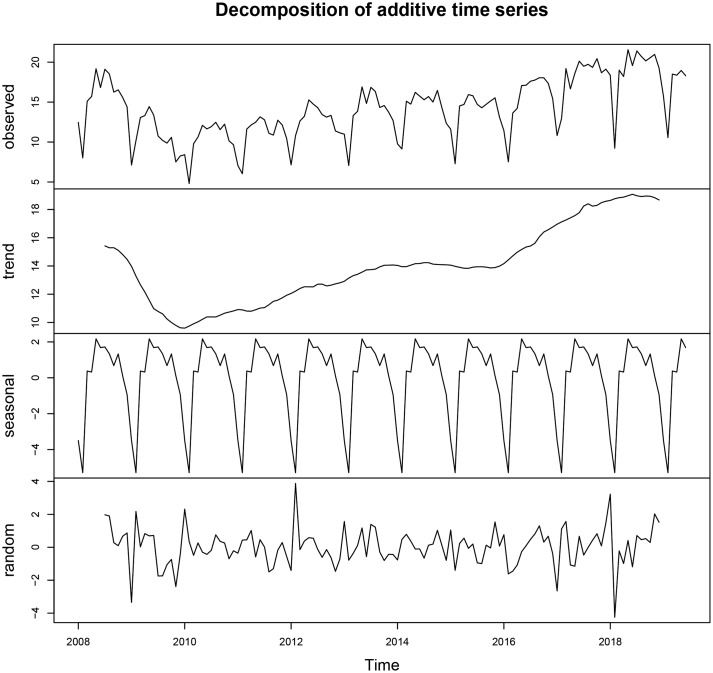
Monthly CT incidence (1/100 000) from January 2008 to June 2019 in Shenzhen and long-term trend, seasonal fluctuation and random fluctuation. (a) The actual CT incidence from January 2008 to June 2019; (b) the decomposed trend trait of CT incidence; (c) the decomposed seasonal trait of CT incidence and (d) the decomposed random fluctuation trait of CT incidence.

**Fig. 2.**
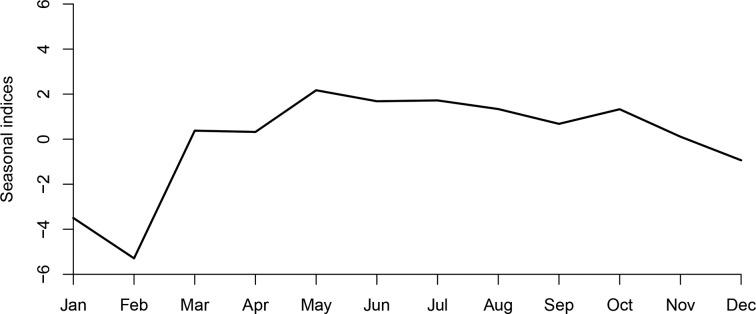
Seasonal indices of CT incidence from January to December in Shenzhen. It can be seen that the incidence of CT mostly peaked in May, and reached the trough in January and February.

The monthly incidence of CT infection ranged from 4.80/100 000 to 21.56/100 000. Figure 1 shows the monthly CT incidence (1/100 000), long-term trend, seasonal fluctuation and random fluctuation in Shenzhen using decomposition methods. There was a slight rising trend from 2010 to 2019 and the seasonal fluctuation indicated that the incidence of CT infection mostly peaked in May, and reached the trough in January and February (Fig. 2).

### SARIMA model

**Fig. 3.**
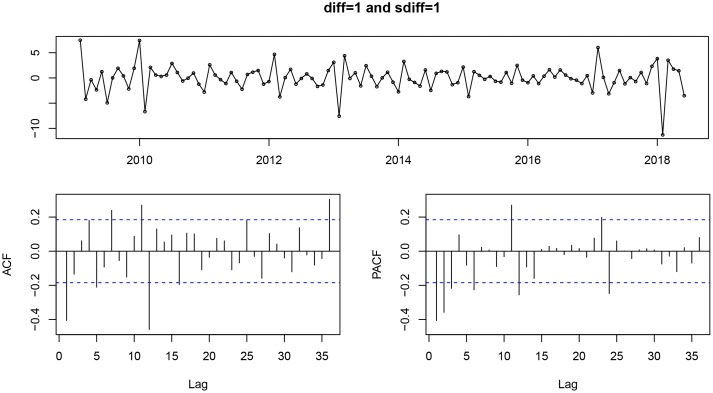
The standardised residual plot (a), ACF (b) and PACF (c) of the series after a first-order non-seasonal difference and first-order seasonal difference. ACF, autocorrelation function; PACF, partial autocorrelation functions.

**Fig. 4.**
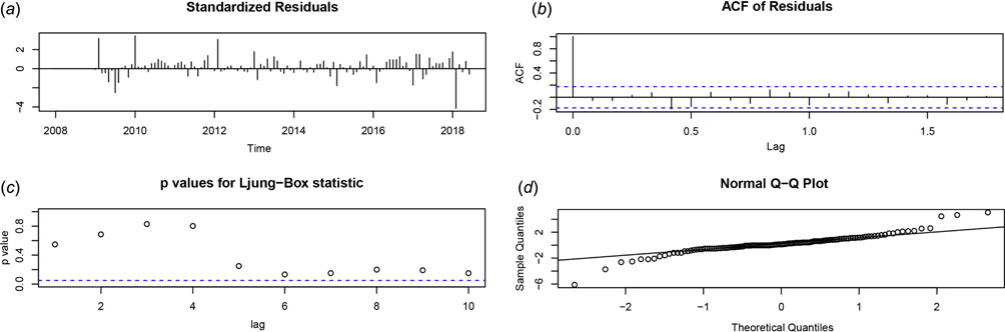
SARIMA (0.1,1)(0.1,1)_12_ model diagnosis. (a) Standardised residual plot; (b) ACF of the errors at various lags; (c) *P* values for Ljung–Box statistic and (d) normal *Q–Q* plot. SARIMA, seasonal autoregressive integrated moving average; ACF, autocorrelation function.

**Fig. 5.**
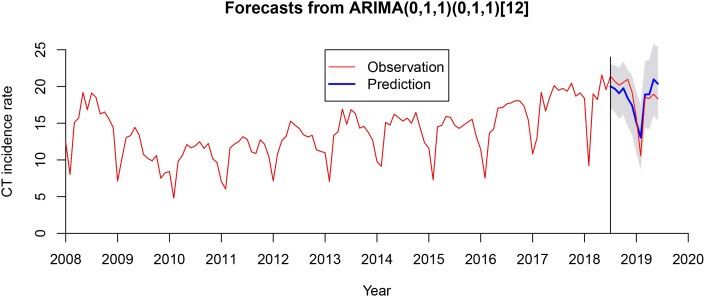
Actual CT incidence from January 2008 to June 2019 in Shenzhen and predicted CT incidence of the SARIMA (0.1,1)(0.1,1)_12_ model from July 2018 to June 2019. The observed values and predicted values of the SARIMA (0.1,1) (0.1,1)_12_ model matched well with the actual incidence falling within the predicted 95% confidence interval (CI). SARIMA, seasonal autoregressive integrated moving average.

After using a first-order non-seasonal difference (*d* = 1) and a first-order seasonal difference (*D* = 1, *s* = 12), the result of the ADF test (*p* = 0.01) showed that the ACF and PACF of the new data tended to be stationary, as also shown in Figure 3. To diagnose the fitness of the model, following criteria should be satisfied: (1) the residuals were distributed with a mean of zero and a constant variance in the standardised residuals; (2) there was no significant deviation from a zero mean white noise process in the ACF of the residuals; (3) the *P* value for the Ljung–Box statistic was greater than 0.05, which means that the null hypothesis of independence for this residual series cannot be rejected and (4) the normal *Q*–*Q* plot of the residuals of the model was normal distributed. We compared AIC values and SBC values of 40 models (Supplementary Table S1) and the SARIMA (0.1,1)(0.1,1)_12_ model was selected as the optimal model with the lowest SBC (SBC = 444.26) and relatively low AIC (AIC = 436.08). Estimated parameters and the Ljung–Box test of the optimal model are shown in Table 1 and all the parameters in the SARIMA (0.1,1)(0.1,1)_12_ model were statistically significant. Besides, the results from the Ljung–Box tests (*Q* = 0.358, *P* = 0.549) indicated that the residual series of these models belong to white noise. Figure 4 shows the graphical diagnostics for assessing the SARIMA (0.1,1) (0.1,1)_12_ model fit, and it fitted well according to the above criteria. Figure 5 shows that the observed values and predicted values of the SARIMA (0.1,1) (0.1,1)_12_ model matched well, with the actual incidence falling within the predicted 95% CI. The equation of the SARIMA (0.1,1) (0.1,1)_12_ model was shown as *y_t_* = ((1 + 0.634B) × (1 + 0.867B^12^) × *ɑ_t_*)/((1 − B) × (1 − B)^12^).

**Table 1.** Estimated parameters and the Ljung–Box test in the optimal SARIMA model

Parameters	Coefficient	Standard error	*T*	*P* value	Residuals of SARIMA model	
					Ljung–Box Q	*P* value
SARIMA (0.1,1)(0.1,1)_12_					0.358	0.549
MA(1)	−0.634	0.072	−8.860	<0.001		
SMA(1)	−0.867	0.029	−29.582	<0.001		

SARIMA, seasonal autoregressive integrated moving average; MA1, moving average, lag1; SMA1, seasonal moving average, lag1.

### Performance of the optimal model

The simulating power and the predictive power of the optimal model are shown in Table 2. The MAPE between the predicted values and the actual values was 8.08% in the hold-out period. The predicted values from July 2018 to June 2019 are shown in Table 3. The relative error in February 2019 was relatively high but the actual incidence in February 2019 still fell within the predicted 95% confidence interval.

**Table 2.** In-sample fitting and out-of-sample predicting performance in the optimal model

	Simulating power				Predictive power
Model	MAE	ME	MAPE	RMSE	MAPE
SARIMA (0.1,1)(0.1,1)_12_	0.910	0.239	0.075	1.384	0.081

SARIMA, seasonal autoregressive integrated moving average; MAE, mean absolute error; ME, mean error; MAPE, mean absolute percentage error; RMSE, root mean square error.

**Table 3.** Predicted chlamydia trachomatis incidence from July 2018 to June 2019 with the selected model

Time	Actual incidence	Predicted incidence	Relative error (%)	LCL	UCL
Jul 2018	21.40	20.02	6.45	17.04	22.99
Aug 2018	20.72	19.70	4.92	16.48	22.91
Sep 2018	20.16	19.07	5.41	15.63	22.51
Oct 2018	20.56	19.77	3.84	16.12	23.42
Nov 2018	20.97	18.40	12.26	14.55	22.26
Dec 2018	19.26	17.42	9.55	13.38	21.46
Jan 2019	15.71	15.12	3.76	10.90	19.34
Feb 2019	10.56	13.04	23.48	8.65	17.44
Mar 2019	18.51	18.92	2.22	14.36	23.48
Apr 2019	18.37	18.93	3.05	14.21	23.64
May 2019	18.96	20.97	10.60	16.10	25.85
Jun 2019	18.30	20.36	11.26	15.34	25.39

SARIMA, seasonal autoregressive integrated moving average; LCL, lower confidence limit; UCL, upper confidence limit.

## Discussion

Case reporting is one of core components of Sexually Transmitted Infections (STI) surveillance [28], which could provide the magnitude of STI burden in general population (the incidence of new infections). The high quality data through a good predictive model could reflect trends in sexual transmission and the effectiveness of STI efforts [29], and thus helping integrate STI surveillance and implement relevant programmes more effectively and precisely. CT is an important public health issue globally and it is important to have a better understanding of the magnitude of future CT burden in the general population. To our knowledge, this is the first study to apply a SARIMA model to fit and predict the incidence trend of CT infection.

In our study, the optimal SARIMA model showed highly accurate forecasting performance (MAPE = 8.08%), with their MAPE value falling within 5%–10% [30]. As a practical and low-cost method with only collecting a time variable, this SARIMA model could provide precise estimates of future CT incidence and its future trend, and also provide a theoretical basis for the development of targeted interventions for disease control and prevention. Also, this SARIMA model could provide early warning to health authorities to have advanced plan and timely implement relevant STI control strategies. Besides, a good prediction of CT incidence may help the evaluation of future interventions. For example, after promoting routine CT screening in a city, using the real-time predicted values from a model with good performance to evaluate the expansion of CT screening may be better than using those actual but old values.

An obvious seasonal fluctuation of CT incidence from 2008 to 2019 was also found in the present study. Specifically, the seasonal fluctuation showed that the CT incidence in Shenzhen decreased from December to February each year, with the trough in February, but later increased. The result was similar to previous studies on other sexually transmitted diseases such as gonorrhea and syphilis in China [31–33], which could be interpreted by what is called ‘Spring Festival effects’ [34]. The ‘Spring Festival effects’ refer to the fact that the number of new infections dropped during the Spring Festival, followed by a rise [32, 34].

Shenzhen is a developed city with large amounts of rural-to-urban migrants. This population is reported to be more likely to engage in high-risk sexual behaviour and more vulnerable to sexually transmitted diseases [31, 35]. Every year before the Spring Festival, many migrant workers return to their rural hometown from about November, which leads to a reduction in sexually active population and reported cases of CT, and thereby a decrease of reported CT incidence in Shenzhen [32]. Additionally, many suspected cases would not like to go to clinics for examination during the festival, also probably contributing to the decline of reported incidence [31]. With the return of migrant workers to Shenzhen since early March, the incidence of CT infection rose again.

The findings about ‘Spring Festival effects’ have important implications for the design of future programmes targeting on this specific population. First, health access in urban areas is usually better than that in rural areas. The ‘Spring Festival effects’ did not only affect case-reporting of CT but also result in the missed opportunity of case management for those patients who return to their hometowns. Additionally, these patients may bring the infections home to further transmit to their couples or sexual partners. A survey in southwest China indicates a higher prevalence of syphilis among left-behind women (1.5%) in rural areas than the general population [36], which suggests that this high-risk group should be targeted in rural intervention programmes. For example, education about STI-related symptoms can be conducted in rural areas to promote their awareness of seeking medical attention. Second, localised outbreaks of CT infection in Shenzhen may be expanded by this floating population [37]. Therefore, due to the peak in May and the rising after February in the CT incidence, rural-to-urban migrants should be targeted in the prevention and intervention programmes such as sexual health promotion to avoid risky sexual behaviours before this population return to their hometown, and the government needs to pay more attention to the screening of CT among them. For instance, prevention publicity for CT can be implemented in crowd gathering place such as long-distance bus stations and train stations.

It was noteworthy that there was a slightly rising trend of CT incidence since 2010. One possible reason is that laboratory tests with higher performance such as the polymerase chain reaction and point-of-care tests were increasingly used, which could help detect more CT cases than using microscope and antigen detection. Second, the enhanced awareness of asymptomatic CT cases by physicians could also help detect more infected cases.

There were some limitations in the study to be considered. First, in the SARIMA model, stationarity of the time series is necessary before fitting and updating the model. Second, the SARIMA model can only be applied to short-term prediction. New observation series should be continually added over time to adjust the model to ensure the prediction accuracy. Furthermore, the performance of the model was also affected by the quality of surveillance data. Enhanced consciousness of seeking treatment in different medical institutions would lead to duplicated reported cases in the system, which was hard to explore the effect.

In summary, the SARIMA model could be applied to effectively predict the short-term CT incidence in Shenzhen, which contributed to a better insight into the future epidemic trends of CT infection. We suggested that the models could be used as a reminder for policy-makers to allocate health resources reasonably and formulate preventive and control programmes for CT infection timely.

## Supplementary material

For supplementary material accompanying this paper visit https://doi.org/10.1017/S0950268820000680. click here to view supplementary material

## References

[1] Rowley J (2019) Chlamydia, gonorrhoea, trichomoniasis and syphilis: global prevalence and incidence estimates, 2016. Bulletin of the World Health Organization 97, 548–562. 31384073 10.2471/BLT.18.228486 PMC6653813

[2] Westrom L (1992) Pelvic inflammatory disease and fertility. A cohort study of 1844 women with laparoscopically verified disease and 657 control women with normal laparoscopic results. Sexually Transmitted Diseases 19, 185–192. 1411832

[3] Haggerty CL (2010) Risk of sequelae after Chlamydia trachomatis genital infection in women. Journal of Infectious Diseases 201(Suppl 2), S134–S155. 20470050 10.1086/652395

[4] Taylor BD and Haggerty CL (2011) Management of Chlamydia trachomatis genital tract infection: screening and treatment challenges. Infection and Drug Resistance 4, 19–29. 21694906 10.2147/IDR.S12715 PMC3108753

[5] Joki-Korpela P (2009) The role of Chlamydia trachomatis infection in male infertility. Fertility and Sterility 91, 1448–1450. 18706556 10.1016/j.fertnstert.2008.06.051

[6] Zhu H (2016) Chlamydia trachomatis infection-associated risk of cervical cancer: a meta-analysis. Medicine (Baltimore) 95, e3077. 27043670 10.1097/MD.0000000000003077 PMC4998531

[7] Castellsague X (2014) Prospective seroepidemiologic study on the role of human papillomavirus and other infections in cervical carcinogenesis: evidence from the EPIC cohort. International Journal of Cancer 135, 440–452. 24338606 10.1002/ijc.28665

[8] Owusu-Edusei JK (2013) The estimated direct medical cost of selected sexually transmitted infections in the United States, 2008. Sexually Transmitted Diseases 40, 197–201. 23403600 10.1097/OLQ.0b013e318285c6d2

[9] Wu JB, Ye LX and You EK (2007) Prediction of incidence of notifiable contagious diseases by application of time series model. Journal of Mathematical Medicine **1**, 90–92.

[10] Zeng Q (2016) Time series analysis of temporal trends in the pertussis incidence in Mainland China from 2005 to 2016. Scientific Reports 6, 32367. 27577101 10.1038/srep32367 PMC5006025

[11] Wang K (2016) The use of an autoregressive integrated moving average model for prediction of the incidence of dysentery in Jiangsu, China. Asia-Pacific Journal of Public Health 28, 336–346. 27106828 10.1177/1010539516645153

[12] Xu Q (2017) Forecasting the incidence of mumps in Zibo city based on a SARIMA model. International Journal of Environmental Research and Public Health 14, 925. 10.3390/ijerph14080925 PMC5580627 28817101

[13] Sun ZQ (2014) Medical Statistics. Beijing: People's Medical Publishing House.

[14] Martinez EZ, Silva EA and Fabbro AL (2011) A SARIMA forecasting model to predict the number of cases of dengue in Campinas, State of Sao Paulo. Brazil. Revista da Sociedade Brasileira de Medicina Tropical 44, 436–440. 21860888 10.1590/s0037-86822011000400007

[15] Zheng YL (2015) Forecast model analysis for the morbidity of tuberculosis in Xinjiang, China. PLoS One 10, e116832. 10.1371/journal.pone.0116832 PMC4356615 25760345

[16] Peng Y (2017) Application of seasonal auto-regressive integrated moving average model in forecasting the incidence of hand-foot-mouth disease in Wuhan, China. Journal of Huazhong University of Science and Technology-Medical Sciences 37, 842–848. 10.1007/s11596-017-1815-8 29270741

[17] Song X (2016) Time series analysis of influenza incidence in Chinese provinces from 2004 to 2011. Medicine (Baltimore) 95, e3929. 27367989 10.1097/MD.0000000000003929 PMC4937903

[18] Box GEP (2015) Time Series Analysis: Forecasting and Control. Journal of the Operational Research Society 22, 199–201.

[19] Chen L (2016) Epidemiological survey of sexually transmitted diseases in Guangdong province in 2014. Journal of Diagnosis and Therapy on Dermato-venereology 23, 3–7.

[20] National Bureau Of Statistics Of China (2015) China Statistical Yearbook 2015. Beijing, China: China Statistic Press.

[21] National Health Commission Of The People'S Republic Of China (2016) Diagnostic of genital chlamydia trachomatis infection. *In WS/T 513-2016*.

[22] Ho SL, Xie M and Goh TN (2002) A comparative study of neural network and Box-Jenkins ARIMA modeling in time series prediction. Computers & Industrial Engineering 42, 371–375.

[23] Galbraith J and Zinde-Walsh V (1999) On the distributions of Augmented Dickey–Fuller statistics in processes with moving average components. Journal of Econometrics 93, 25–47.

[24] Koehler A and Murphree ES (1988) A comparison of the Akaike and Schwarz criteria for selecting model order. Applied Statistics **37**, 187.

[25] Mer Faruk D (2010) A hybrid neural network and ARIMA model for water quality time series prediction. Engineering Applications of Artificial Intelligence 23, 586–594.

[26] Ljung G and Box G (1978) On a Measure of Lack of Fit in Time Series Models. Biometrika 65, 297–303.

[27] Fong PW and Li WK (2003) On time series with randomized unit root and randomized seasonal unit root. Computational Statistics & Data Analysis 43, 369–395.

[28] UNAIDS/WHO Working Group on Global HIV/AIDS/STI Surveillance (2012) Strategies and Laboratory Methods for Strengthening Surveillance of Sexually Transmitted Infection 2012. Geneva, Switzerland: World Health Organization, ISBN 9789241504478.

[29] World Health Organization (2015) A Tool for Strengthening STI Surveillance at the Country Level. Geneva, Switzerland: World Health Organization, WHO/RHR/15.06.

[30] Wang Y (2019) Temporal trends analysis of tuberculosis morbidity in mainland China from 1997 to 2025 using a new SARIMA-NARNNX hybrid model. BMJ Open 9, e24409. 10.1136/bmjopen-2018-024409 PMC6678063 31371283

[31] Zhang X (2016) Time series modelling of syphilis incidence in China from 2005 to 2012. PLoS One 11, e149401. 10.1371/journal.pone.0149401 PMC4763154 26901682

[32] Wu QH (2018) Epidemiological characteristics of gonorrhea from 2005 to 2016 and ARIMA model for predicting the incidence trend in Nanshan District of Shenzhen. Chinese Journal of Infection Control 17, 202–206.

[33] Tan NX (2014) Temporal trends in syphilis and gonorrhea incidences in Guangdong province, China. Journal of Infectious Diseases 209, 426–430. 24041788 10.1093/infdis/jit496 PMC3883174

[34] Wei S (2013) “Spring Festival effects” on the main notifiable communicable diseases in China. Fudan University Journal of Medical Sciences 40, 153–158.

[35] Zhang L (2009) Building a Better Infectious Disease Surveillance System for China: An Evaluation from a Political Perspective. Saarbrücken, Germany: VDM Verlag Dr. Müller, ISBN 978-3-639-19654-2.

[36] Zhong J, Tan L and Wang G (2010) Analysis of syphilis and AIDS infection in 200 left-behind women in rural areas of Wuzhou City. Journal of Preventive Medicine Information 26, 998.

[37] Tucker JD and Cohen MS (2011) China's syphilis epidemic: epidemiology, proximate determinants of spread, and control responses. Current Opinion in Infectious Diseases 24, 50–55. 21150594 10.1097/QCO.0b013e32834204bf PMC3103765

