# Supplementary material for: Time series analysis and forecasting of chlamydia trachomatis incidence using surveillance data from 2008 to 2019 in Shenzhen, China
Source: Epidemiol Infect. 2020 Mar 17;148:e76. doi: 10.1017/S0950268820000680 (PMC7163807; doi:10.1017/S0950268820000680)
Supplement: Supplementary file 1 [file S0950268820000680sup001.docx]

*Epidemiology and Infection*

**Time series analysis and forecasting of chlamydia trachomatis incidence using surveillance data from 2008 to 2019 in Shenzhen, China**

**Authors**

R. X. WENG^1†^, H. L. FU^1,2†^, C. L. ZHANG^1^, J. B. YE^1^, H. L. WANG^1^, F. C. HONG^1^, X. S. CHEN^3,4,5*^, Y. M. CAI^1*^

Supplementary Material

**Supplementary Table S1** The AIC and SBC value of all SARIMA models

| Models | AIC | SBC | Models | AIC | SBC |
| --- | --- | --- | --- | --- | --- |
| SARIMA (0,1,1)(0,1,1)_12_ | 436.08 | 444.26 | SARIMA (0,1,1)(0,1,0)_12_ | 494.30 | 499.76 |
| SARIMA (1,1,1)(0,1,1)_12_ | 436.96 | 447.87 | SARIMA (1,1,1)(0,1,0)_12_ | 496.30 | 504.48 |
| SARIMA (2,1,1)(0,1,1)_12_ | 437.51 | 451.15 | SARIMA (2,1,1)(0,1,0)_12_ | 495.84 | 506.75 |
| SARIMA (3,1,1)(0,1,1)_12_ | 436.64 | 453.00 | SARIMA (3,1,1)(0,1,0)_12_ | 493.97 | 507.61 |
| SARIMA (6,1,1)(0,1,1)_12_ | 438.56 | 463.11 | SARIMA (6,1,1)(0,1,0)_12_ | 491.28 | 513.10 |
| SARIMA (0,1,0)(0,1,1)_12_ | 473.33 | 478.79 | SARIMA (0,1,0)(0,1,0)_12_ | 533.25 | 535.97 |
| SARIMA (1,1,0)(0,1,1)_12_ | 446.08 | 454.26 | SARIMA (1,1,0)(0,1,0)_12_ | 512.79 | 518.25 |
| SARIMA (2,1,0)(0,1,1)_12_ | 437.21 | 448.12 | SARIMA (2,1,0)(0,1,0)_12_ | 497.74 | 505.92 |
| SARIMA (3,1,0)(0,1,1)_12_ | 435.91 | 449.55 | SARIMA (3,1,0)(0,1,0)_12_ | 493.54 | 504.45 |
| SARIMA (6,1,0)(0,1,1)_12_ | 436.81 | 458.63 | SARIMA (6,1,0)(0,1,0)_12_ | 489.45 | 508.54 |
| SARIMA (0,1,5)(0,1,1)_12_ | 438.69 | 457.78 | SARIMA (0,1,5)(0,1,0)_12_ | 491.21 | 507.58 |
| SARIMA (1,1,5)(0,1,1)_12_ | 440.34 | 462.16 | SARIMA (1,1,5)(0,1,0)_12_ | 492.37 | 511.46 |
| SARIMA (2,1,5)(0,1,1)_12_ | 433.25 | 457.80 | SARIMA (2,1,5)(0,1,0)_12_ | 489.46 | 511.28 |
| SARIMA (3,1,5)(0,1,1)_12_ | 435.13 | 462.40 | SARIMA (3,1,5)(0,1,0)_12_ | 490.77 | 515.31 |
| SARIMA (6,1,5)(0,1,1)_12_ | 439.04 | 474.49 | SARIMA (6,1,5)(0,1,0)_12_ | 478.11 | 510.83 |
| SARIMA (0,1,7)(0,1,1)_12_ | 438.29 | 462.83 | SARIMA (0,1,7)(0,1,0)_12_ | 478.83 | 500.65 |
| SARIMA (1,1,7)(0,1,1)_12_ | 440.22 | 467.49 | SARIMA (1,1,7)(0,1,0)_12_ | 480.57 | 505.12 |
| SARIMA (2,1,7)(0,1,1)_12_ | 442.22 | 472.22 | SARIMA (2,1,7)(0,1,0)_12_ | 481.40 | 508.67 |
| SARIMA (3,1,7)(0,1,1)_12_ | 438.95 | 471.67 | SARIMA (3,1,7)(0,1,0)_12_ | 483.38 | 513.39 |
| SARIMA (6,1,7)(0,1,1)_12_ | 442.34 | 483.25 | SARIMA (6,1,7)(0,1,0)_12_ | 474.48 | 512.66 |

***Abbreviation*:** AIC= Akaike Information Criterion, SBC= Schwartz Bayesian Criterion.
